# Mass Cytometry Reveals a Sustained Reduction in CD16^+^ Natural Killer Cells Following Chemotherapy in Colorectal Cancer Patients

**DOI:** 10.3389/fimmu.2019.02584

**Published:** 2019-11-05

**Authors:** Diana Shinko, Helen M. McGuire, Connie I. Diakos, Nick Pavlakis, Stephen J. Clarke, Scott N. Byrne, Kellie A. Charles

**Affiliations:** ^1^Discipline of Pharmacology, Faculty of Medicine and Health, The University of Sydney, Sydney, NSW, Australia; ^2^Discipline of Pathology, Faculty of Medicine and Health, The University of Sydney, Sydney, NSW, Australia; ^3^Ramaciotti Facility for Human Systems Biology, The University of Sydney, Sydney, NSW, Australia; ^4^Northern Sydney Cancer Centre, Royal North Shore Hospital, St Leonards, NSW, Australia; ^5^Faculty of Medicine and Health, Northern Clinical School, The University of Sydney, Sydney, NSW, Australia; ^6^Bill Walsh Translational Research Laboratories, Kolling Institute of Medical Research, St Leonards, NSW, Australia; ^7^Discipline of Infectious Diseases and Immunology, Faculty of Medicine and Health, The University of Sydney, Sydney, NSW, Australia; ^8^Westmead Institute for Medical Research, Centre for Immunology and Allergy Research, Westmead, NSW, Australia

**Keywords:** mass cytometry, colorectal cancer, NK cells, chemotherapy, signalling pathways

## Abstract

The immune system and inflammation plays a significant role in tumour immune evasion enhancing disease progression and reducing survival in colorectal cancer (CRC). Patients with advanced stages of colorectal cancer will all undergo treatment with cytotoxic chemotherapy which may alter the complexity of immune cell populations. This study used mass cytometry to investigate the circulating immune cell profile of advanced CRC patients following acute and chronic doses of standard cytotoxic chemotherapy and analysed seven major immune cell populations and over 20 subpopulations. Unsupervised clustering analysis of the mass cytometry data revealed a decrease in NK cells following one cycle of cytotoxic chemotherapy. Investigation into the NK sub-population revealed a decline in the CD56^dim^ CD16^+^ NK cell population following acute and chronic chemotherapy treatment. Further analysis into the frequency of the NK cell sub-populations during the long-term chemotherapy treatment revealed a shift in the sub-populations, with a decrease in the mature, cytotoxic CD56^dim^ CD16^+^ accompanied by a significant increase in the less mature CD56^dim^ CD16^−^ and CD56^bright^ NK cell populations. Furthermore, analysis of the phosphorylation status of signalling responses in the NK cells found significant differences in pERK, pP38, pSTAT3, and pSTAT5 between the patients and healthy volunteers and remained unchanged throughout the chemotherapy. Results from this study reveals that there is a sustained decrease in the mature CD16^+^ NK cell sub-population frequency following long-term chemotherapy which may have clinical implications in therapeutic decision making.

## Introduction

An estimated 1.4 million people are annually diagnosed with colorectal cancer (CRC) making it the third most common cancer in the world (1). Despite many nations operating screening programs, over half the people present with advanced or metastatic disease requiring chemotherapy (1). Therapeutic decisions for CRC patients are highly dependent on the staging and the site of the tumour. For people with locally advanced or metastatic CRC, combination cytotoxic chemotherapy is the backbone to all chemotherapy regimens, which primarily involves 5-fluorouracil (5-FU), folinic acid (leucovorin), and either oxaliplatin (FOLFOX) or irinotecan (FOLFIRI) (2, 3). With the advances in molecular targeted therapies, metastatic CRC has benefited from the addition of molecular targeted drugs, such as EGFR or VEGF inhibitors, cetuximab and bevacuzimab, respectively (4, 5). These targeted agents have extended the median long-term survival to almost 3 years for people with metastatic CRC (4). Further improvements in survival will require new drug targets to be identified.

A growing understanding of the various hallmarks of cancer has meant that new drivers of carcinogenesis can be identified and exploited (6). A characteristic feature of colorectal cancer is the complex role that the immune system and inflammation plays in tumour immune evasion and tumour progression (7). Interactions between inflammatory cytokines, immune cells, angiogenesis and malignant epithelial tumour cells may provide novel drug targets for cancer treatment (8, 9). While targeting the immune system with immune checkpoint inhibitors has had a staggering impact on some cancers, immunotherapy has not been as effective in the treatment of CRC (10). Indeed, check point inhibitors appear only to be effective in people with CRC tumours that contain a mismatch-repair deficiency (~15% of all CRC tumours) (11, 12). The development of new immunotherapies for CRC will require a more detailed understanding of the immune cells involved in CRC carcinogenesis, particularly during standard cytotoxic chemotherapy.

Mass cytometry is a multi-parametric technique that offers unparalleled insight into functional and biological systems, including immune cells, at the single cell level (13). Mass cytometry overcomes some of the deficiencies of fluorescence flow cytometry by utilising stable, heavy metal isotopes as tags that can be conjugated to over 100 membrane and intracellular targets on single cells in the same sample (13, 14). Newly developed unsupervised clustering algorithms have enabled users to move away from directed analysis of this high-dimensional data towards identification of underappreciated sub-populations (15).

This study used mass cytometry to investigate the circulating immune cell profile of advanced CRC patients undergoing chemotherapy. Our mass cytometry analysis revealed a distinct phenotypic shift in Natural Killer (NK) cell phenotype following chemotherapy treatment, with a significant decrease in CD56^dim^ CD16^+^ cells and a significant increase in CD56^dim^ CD16^−^ and CD56^bright^ cells. Furthermore, the use of mass cytometry allowed the examination of the phosphorylation status of signalling responses in the NK cells and found significant differences in pERK, pP38, pSTAT3, and pSTAT5 between the CRC patients and the healthy volunteers.

## Materials and Methods

### Patient Samples

Ten patients with stage III or IV colorectal cancer (CRC) undergoing chemotherapy between 2015 and 2017 at Northern Cancer Institute (St Leonards, NSW, Australia) were recruited to this study (Table 1). Nine age and gender matched healthy volunteers were also recruited. This study was approved by Northern Sydney Local Health District and North Shore Private Hospital Human Ethics Committees, and written, informed consent was obtained from all patients prior to participation in the study.

**Table 1:** Patient demographics.

	**Number**	**Percentage**
CRC patients	10	
**Gender:**		
Male: Female	6:4	60:40
**Age:**		
Median (range)	66 (40–81)	
**Tumour site:**		
Colon, rectum, other	5, 2, 1	50:20:10
**Stage of cancer:**		
III, IV	6, 4	60:40
**Mismatch repair**		
Deficient, proficient	1, 9	10:90
**Treatment**		
FOLFOX, other	10, 0	100:0

### Chemotherapy Treatment and Sample Collection

**Figure 1:**
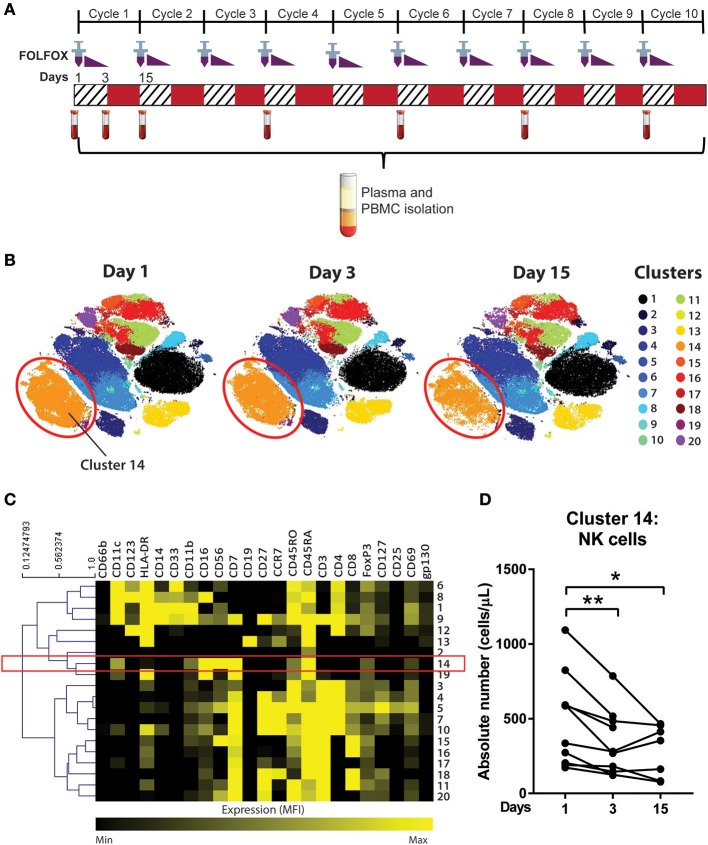
Unsupervised clustering algorithm revealed a decline in NK cell numbers in CRC patients following acute chemotherapy. **(A)** Blood samples were collected from CRC patients undergoing FOLFOX chemotherapy on day 1 (pre-treatment), day 3, and day 15 (pre-cycle 2) of the first cycle of chemotherapy and throughout the later cycles of chemotherapy (cycles 4, 6, 8, and 10). PBMCs were isolated and stained with a 35-antibody mass cytometry panel and ran on the mass cytometer, Helios^TM^. **(B)** Data from samples taken on the first cycle of chemotherapy (days 1, 3, and 15) was analysed using an unsupervised clustering algorithm, FlowSOM, and the 20 clusters were visualised using tSNE plots. **(C)** A heatmap was generated to show expression of the median fluorescence intensity (MFI) of each surface marker for the clusters. **(D)** The absolute number of cluster 14 throughout days 1, 3, and 15 of the chemotherapy was statistically analysed using Wilcoxon test. ^*^p < 0.05, ^**^p < 0.01. *n* = 10.

All patients in this study were treated with the 3 day FOLFOX regimen consisting of 5-FU, oxaliplatin and leucovorin. On day 1, a patient is administered 85 mg/m^2^ oxaliplatin as an intravenous (i.v.) infusion, 50 mg calcium folinate (leucovorin) as i.v. bolus injection, 400 mg/m^2^ of 5-FU as i.v. bolus injection, and 2,400 mg/m^2^ 5-FU as a continuous i.v. infusion via a pump over 46 h (16). Twenty millilitres of blood was collected from patients in K_2_ EDTA-lavender top tubes (BD Bioscience) on day 1 (pre-treatment), day 3, and day 15 (pre-cycle 2 treatment) (Figure 1A). Full blood counts were collected within 24 hours of the research blood collection. Blood samples prior to the start of chemotherapy in cycles 4, 6, 8, 10 of the treatment were also collected while participants remained on active chemotherapy (Figure 1A). Age and gender matched blood samples from a Healthy Volunteer study, approved by the Sydney Local Health District at Royal Prince Alfred Hospital, Camperdown were utilised to compare immune cell phenotypes with the pre-chemotherapy blood samples from the patient cohort.

### Isolation of PBMCs

Peripheral blood mononuclear cells (PBMCs) were isolated using Ficoll-paque density gradient separation (density 1.077 ± 0.003 g/dL; GE healthcare life sciences). Blood was mixed with phosphate buffer saline (PBS), added to a layer of Ficoll-paque reagent and centrifuged at 550 g for 20 min at 22°C, brake off. The layer of PBMCs is then removed and washed twice in PBS through centrifugation (550 g for 5 min at 22°C). PBMCs were resuspended in freezing media (90% foetal bovine serum; FBS and 10% dimethyl sulfoxide; DMSO) and frozen in liquid nitrogen for long term storage.

### Mass Cytometry

The isolated PBMCs were labelled with metal-conjugated antibodies for mass cytometry using an optimised and established protocol (17). The antibodies used were either purchased pre-conjugated from Fluidigm, conjugated and validated in-house or provided by the Ramaciotti Facility for Human Systems Biology (RFHSB) at the University of Sydney. The panel of antibodies used can be found in Table 2 and Supplementary Table 1.

**Table 2:** The antibody panel used for mass cytometry.

**Metal tag**	**Target**	**Clone**
141Pr	CD235ab	HIR2
142Nd	CD19	HIB19
143Nd	CD33	WM53
144Nd	CD11b	ICR544
145Nd	CD4	RPA-T4
146Nd	CD8	RPA-T8
147Sm	CD7	CD7-6B7
148Sm	CD16	3G8
149Sm	CD25	2A3
150Nd	pSTAT5 (Y694)	47
151Eu	CD123	6H6
152Sm	CD66b	80H3
153Eu	pSTAT1 (Y701)	58D6
154Sm	pAKT	J1-223.371
155Gd	CD27	L128
156Gd	pP38 (T180/Y182)	36/p38
158Gd	pSTAT3 (Y705)	4/p
159Tb	CD11c	Bu15
160Gd	CD14	M5E2
161Dy	CD69	FN50
162Dy	FoxP3	PCH101
163Dy	CD56 (NCAM)	HCD56
164Dy	CD45RO	UCHL1
166Er	pP65 (S529)	K10.895.12.50
167Er	CD197 (CCR7)	G043H7
168Er	pERK (T202/Y204)	D13.14.4E
169Tm	CD45RA	HI100
170Er	CD3	UCHT1
171Yb	CD66a/c/e	ASL-32
172Yb	CD130 (gp130)	2E1B02
173Yb	pMAPKAPK2 (Thr334)	27B7
174Yb	HLA-DR	L243
175Lu	Arginase 1	658922
176Yb	CD127 (IL-7Rα)	A019D5

PBMCs were thawed in a 37°C water bath and washed twice with media (RPMI + 10% FBS) and once with CyFACS buffer (0.5% bovine serum albumin; BSA, 0.02% sodium azide, 2 mM ethylenediaminetetraacetic acid; EDTA, in phosphate-buffered saline; PBS) by centrifuging at 500 g for 5 min. A surface antibody master mix (surface marker antibodies diluted in CyFACS buffer) was applied to the samples and incubated for 30 min at 4°C. Samples were washed 3 times with CyFACS buffer by centrifuging at 500 g for 5 min at 4°C. The samples were stained for FoxP3 using the Foxp3/Transcription Factor staining Buffer Set (ebioscience^TM^) according to the manufacturer's instructions. Briefly, the cells were fixed and permeabilisation using the provided fixation/permeabilisation buffer (30–60 min at 22°C). The cells were stained with FoxP3 antibody (diluted in the provided permeabilisation buffer) by incubating for 30 min at 22°C. The samples were washed with permeabilisation buffer and CyFACS buffer by centrifuging at 800 g for 8 min.

The samples were then permeabilised by adding 100% ice-cold methanol to each sample and incubating for 30 min on ice. The samples were washed once with CyFACS buffer and resuspended in a master phospho-antibody mixture (phospho-antibodies diluted in CyFACS buffer) and incubated for 45–60 min at 22°C. The cells were washed 3 times and resuspended in 4% paraformaldehyde and incubated overnight. The samples were washed with CyFACS buffer followed by resuspension in 0.125 μM of the Cell ID^TM^ DNA intercalator-Ir (Fluidigm) and incubated for 20 min at 22°C. The cells were then washed three times with ultrapure water (18 MΩ-cm) and resuspended in normalisation beads (1X EQ^TM^ four element calibration beads; Fluidigm) and filtered through a 35 μm nylon mesh and analysed on a CyTOF 2 Helios^TM^ upgraded mass cytometer (Fluidigm). Normalisation procedure of the generated FCS files were carried out using the CyTOF acquisition software (Fluidigm) based on the concurrently run EQ beads.

### Data Analysis

Analyses were performed using the Cytometry Analysis Pipeline for large and complex datasets (CAPX), a workflow for discovery of high-dimensional cytometry data providing a set of scripts using existing clustering and visualisation tools (18, 19). The pipeline uses the clustering algorithm, FlowSOM, an R package from Bioconductor, and visualisation using a t-Distributed Stochastic Neighbour Embedding (tSNE) plots. The data was pre-gated using FlowJo v10.4.1 (TreeStar, Inc.) on DNA^+^, Single cells, CD235ab^−^ CD66a/c/e^−^ population and exported for further analysis (Supplementary Figure 1). The pre-processed samples were then clustered using the CAPX pipeline which was performed using RStudio. Cells were clustered on expression of lineage markers CD11b, CD11c, CD123, CD127, CD14, CD16, CD19, CD197 (CCR7), CD25, CD27, CD3, CD33, CD4, CD45RA, CD45RO, CD56, CD7, CD8, HLA-DR. An equal number of events were sampled without replacement from all files.

**Figure 2:**
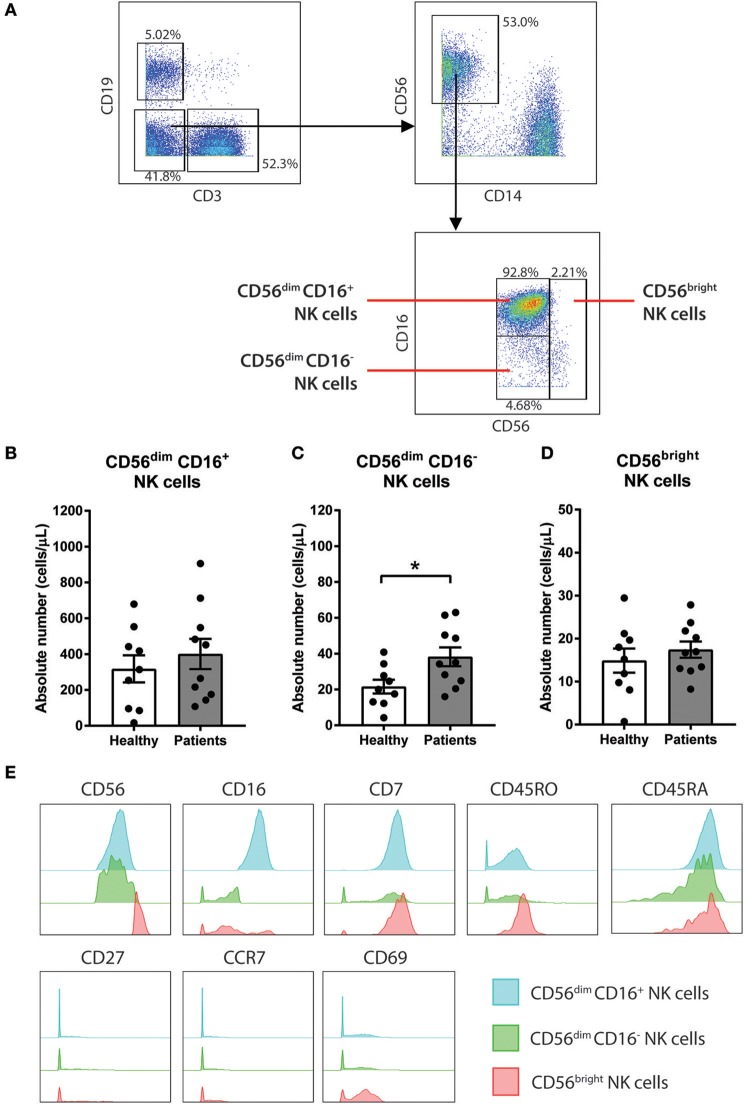
CD56^dim^ CD16^−^ NK cells are increased in baseline of CRC patients compared to healthy volunteers. **(A)** The gating strategy used to identify the 3 sub-population of NK cells. CD3^−^ CD19^−^ population was gated out first followed by gating on the CD14^−^ CD56^+^ total NK cell population. The subtypes of NK cells were gated based on expression of CD56 and CD16 to characterise 3 sub-populations CD56^dim^ CD16^+^, CD56^dim^ CD16^−^, and CD56^bright^. The differences in **(B)** CD56^dim^ CD16^+^, **(C)** CD56^dim^ CD16^−^, and **(D)** CD56^bright^ NK cells between the CRC patients pre-treatment compared to the healthy volunteers was statistically analysed using Mann-Whitney *U*-test. **(E)** The expression levels of various surface markers in each NK cell sub-population is visualised in a modal histogram. ^*^p < 0.05, *n* = 19.

The mass cytometry results were also further analysed using a manual gating strategy on FlowJo. All samples underwent the same background gating strategy mentioned above (Supplementary Figure 1). Further gating strategies for NK cell subtypes are described in Figure 2. The frequency of cell populations was determined as a percentage of parent population. Absolute numbers of cell population was determined using the frequency as a percentage of PBMC population and the lymphocyte or monocyte counts. For instance, the absolute cell number of NK cells was measured using the formula:

$$\begin{array}{l} \frac{frequency\;of\;cells\;\left(\%\;of\;total\;PBMCs\right)}{frequncy\;of\;total\;lymphocytes\;\left(\%\;of\;total\;PBMCs\right)}\;x\;lymphocyte\;count\;\left(cells/\mu L\right) \end{array}$$

### Statistical Analysis

Statistical analysis was performed using GraphPad Prism 7.0. All data was statistically analysed using non-parametric tests. Mann-Whitney *U*-test was used for un-paired data including comparing healthy volunteers to patients. Wilcoxon test was used for paired data. In the cases of any missing data points (such as missing day 3 samples), only full pairs were used in the paired Wilcoxon test. *P* values <0.05 were considered significant. Multiple comparison testing was not performed as the analyses were exploratory in nature and statistical results are to be viewed as hypothesis generating.

## Results

### NK Cell Numbers Decline in CRC Patients Following Acute Chemotherapy

With the development of newer high dimensional analysis techniques, the data was analysed using an unsupervised, automated data clustering analysis; FlowSOM. FlowSOM is a clustering algorithm that analyses the data using self-organising maps based on the similarities of the marker expression between individual cells, followed by hierarchical consensus meta-cluster to merge cells into distinct clusters (20).

To determine the effect of an acute dose of cytotoxic chemotherapy on the immune cell populations, a clustering analysis was conducted using samples collected on days 1, 3, and 15 of the first cycle of chemotherapy (Figure 1A). We analysed the data into 20 clusters based on the expression of 19 surface markers, with the various clusters visualised using tSNE plots (Figure 1B). The FlowSOM clustering revealed a decrease in cluster 14 between days 1, 3, and 15, which can be seen in cluster size in the tSNE plots (Figure 2B). The expression of the median fluorescence intensity (MFI) of each surface marker for the clusters was visualised in a heatmap (Figure 1C). The heatmap showed that cluster 14 expressed CD56, CD16, and CD7 but lacked the expression of CD14, CD19, and CD3 thus we concluded that this population were NK cells (Figure 1C).

Statistical analysis of the absolute number of cells in cluster 14 showed a significant decrease in the population on day 3 compared to day 1 (358.4 ± 72.4 vs. 521.4 ± 101.4 cells/μL; *p* = 0.0039) and on day 15 compared to day 1 (287.2 ± 65.8 vs. 453.4 ± 126.3 cells/μL; *p* = 0.0469; Figure 1D). Supplementary Figure 2 also shows the statistical difference between days 1, 3, and 15 across all clusters identified by the FlowSOM analysis. Of these populations, cluster 14 was the only population which demonstrated a sustained quantitative change following the first cycle or acute chemotherapy treatment therefore we chose to further investigate this population.

### CD56^dim^ CD16^−^ NK Cells Are Increased in CRC Patients

To further understand the impact of chemotherapy on sub-populations of NK cells, additional analysis of the NK cell population was conducted using a manual gating strategy. Total NK cell population was characterised as the CD3^−^ CD19^−^ CD14^−^ CD56^+^ using the gating strategy in Figure 2A. NK cell sub-populations were further gated based on the relative expression of CD56 and CD16 (Figure 2A). The 3 NK cell sub-populations characterised were CD56^dim^ CD16^+^, CD56^dim^ CD16^−^, and CD56^bright^.

Statistical analysis was performed to determine the differences in the NK cells in the CRC patients at baseline compared to a healthy population. No differences in the absolute number of the total NK cell population were observed between the people with CRC and the healthy volunteers (Supplementary Figure 3A). Additionally, no difference in the absolute number of CD56^dim^ CD16^+^ or CD56^bright^ population was seen between the people with CRC and the healthy volunteers (Figures 2B,D). However, there was an increase in the absolute numbers of CD56^dim^ CD16^−^ population in people with CRC compared to the healthy volunteers (38.22 ± 5.29 vs. 21.6 ± 3.84 cells/μL; *p* = 0.0279; Figure 2C).

The phenotype of the NK cell sub-population was further assessed by examining the expression of various surface markers. As expected, the majority of the CD56^bright^ population expressed low or negative levels of CD16. In addition to CD56 and CD16, the NK cells sub-population expressed CD7, CD45RO, and CD45RA (Figure 2E). The NK cell subtypes also expressed low levels of CD69, particularly the CD56^bright^ population (Figure 2E).

### Decrease in NK Cell Sub-populations Following Chemotherapy Treatment

**Figure 3:**
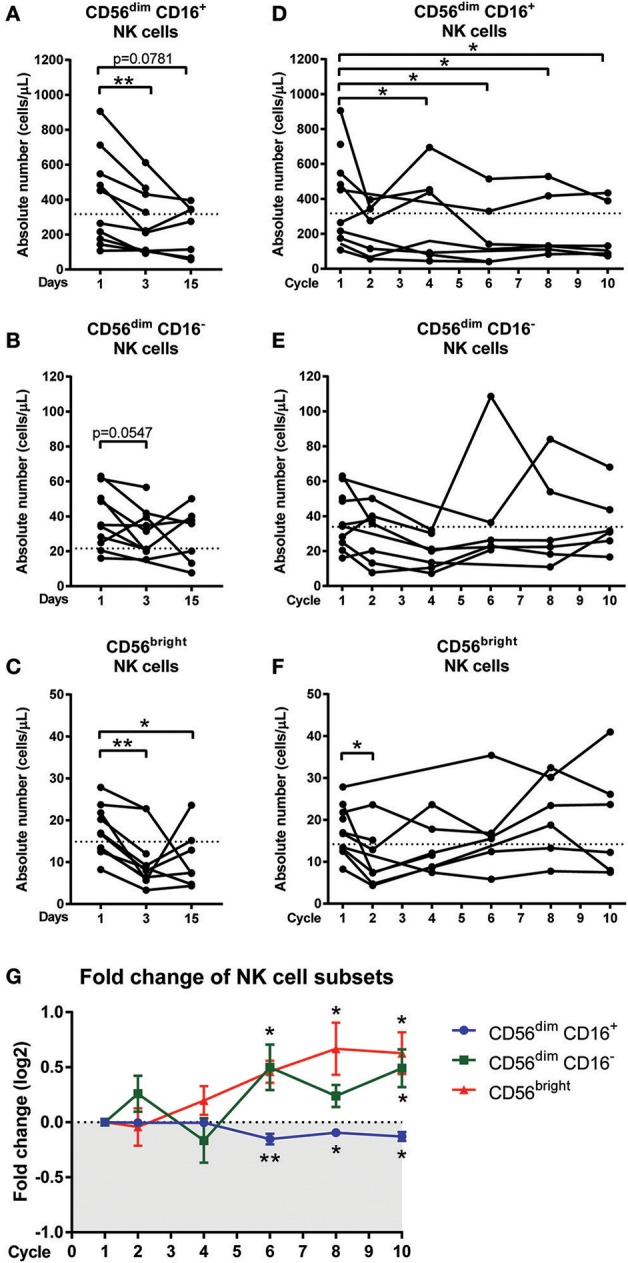
Changes in NK cell sub-populations following chemotherapy treatment. Blood samples collected on day 1 (pre-treatment), day 3, and day 15 of the first cycle in CRC patients undergoing FOLFOX chemotherapy. Blood samples from later cycles were also collected to test the long term effect of chemotherapy. The absolute numbers of **(A)** CD56^dim^ CD16^+^, **(B)** CD56^dim^ CD16^−^, and **(C)** CD56^bright^ NK cells on days 1, 3, and 15 and the absolute numbers of **(D)** CD56^dim^ CD16^+^, **(E)** CD56^dim^ CD16^−^, and **(F)** CD56^bright^ NK cells throughout the later cycles of chemotherapy were statistically analysed using Wilcoxon test. **(G)** The fold change in the frequency of NK cell sub-populations as a percentage of total NK cells over the baseline levels were determined throughout the later cycles of chemotherapy and data presented as mean ± SEM. ^*^p < 0.05, ^**^p < 0.01. *n* = 10.

Manual gating of the total NK cell population confirmed results obtained from the unsupervised clustering approach with a significant decrease in absolute numbers of the total NK cells on days 3 and 15 compared to day 1 (Supplementary Figure 3B). The effect of acute chemotherapy on each of the NK cell sub-population was further examined using manual gating. Results showed a significant decrease in the absolute numbers of CD56^dim^ CD16^+^ population on day 3 compared to day 1 (286.5 ± 35.0 vs. 429.3 ± 88.63 cells/μL; *p* = 0.0078) and a decrease on day 15 compared to day 1, although this did not reach significance (227.9 ± 54.7 vs. 375.2 ± 109.2 cells/μL; *p* = 0.0781; Figure 3A). The CD56^dim^ CD16^−^ population also decreased on day 3 (31.23 ± 4.5 vs. 40.19 ± 5.5 cells/μL; *p* = 0.0547) but no difference was seen on day 15 compared to day 1 (Figure 3B). Results also showed that the CD56^bright^ population significantly decreased on day 3 (10.7 ± 2.4 vs. 18.0 ± 2.0 cells/μL; *p* = 0.0039) compared to day 1. The CD56^bright^ population increased on day 15 but was still significantly lower than the baseline levels on day 1 (10.8 ± 2.6 vs. 16.1 ± 2.0 cells/μL; *p* = 0.0469; Figure 3C).

The effect of long-term chemotherapy treatment on the NK cell populations was also assessed. A decrease in the absolute number of cells in the total NK cell population was observed at cycles 2, 4, 6, and 8 (Supplementary Figure 3C). Results also showed a decrease in the absolute number of cells within the CD56^dim^ CD16^+^ population throughout the chemotherapy treatment, with a significant decrease seen at cycle 4 (280.5 ± 94.2 vs. 368.3 ± 110.6 cells/μL; *p* = 0.0313), cycle 6 (196.2 ± vs. 384.6 ± 122.4 cells/μL; *p* = 0.0313), cycle 8 (233.7 ± 77.4 vs. 395.8 ± 117.7 cells/μL; *p* = 0.0313), and cycle 10 (203.3 ± 66.6 vs. 395.8 ± 117.7 cells/μL; *p* = 0.0313) compared to the baseline levels at cycle 1 (Figure 3D). No significant difference was seen in the absolute numbers of CD56^dim^ CD16^−^ or CD56^bright^ populations through the later cycles of chemotherapy as seen in Figures 3E,F.

In addition to assessing the effect of long-term chemotherapy on the absolute numbers of NK cells, the effect on the frequency of NK cell sub-population was also assessed to investigate any shift in the NK cell sub-population frequency following chemotherapy. Results showed a decline in the CD56^dim^ CD16^+^ population during cycles 6 (*p* = 0.0075), 8, and 10 (*p* = 0.0313) and this is accompanied by increases in CD56^dim^ CD16^−^ at cycle 6 (*p* = 0.0625) and cycle 10 (*p* = 0.0313) and CD56^bright^ sub-population at cycles 6, 8, and 10 (*p* = 0.0313; Figure 3G and Supplementary Figure 4). This result indicated a shift in the NK cell sub-populations, particularly throughout the later cycles of chemotherapy.

### Phosphorylation Signalling Responses in NK Cells of CRC Patients

Inflammatory cytokines and chemokines co-ordinate the recruitment, proliferation, activation, and survival of immune cells around the body to combat infections or inhibit tumour growth that can disrupt homeostasis. When bound to their cognate receptors on immune cells, these mediators will lead to the activation of the inflammatory signalling transcription factors and enable the immune cell to function appropriately in these situations (21, 22).

To assess the activation of the signalling pathways and function of the NK cells, we next investigated the phosphorylation status of 8 signalling proteins. Results showed significant changes in phosphorylation status between the CRC patients compared to healthy controls but chemotherapy did not significantly impact the signalling responses in any NK cell sub-population (Supplementary Figure 5).

**Figure 4:**
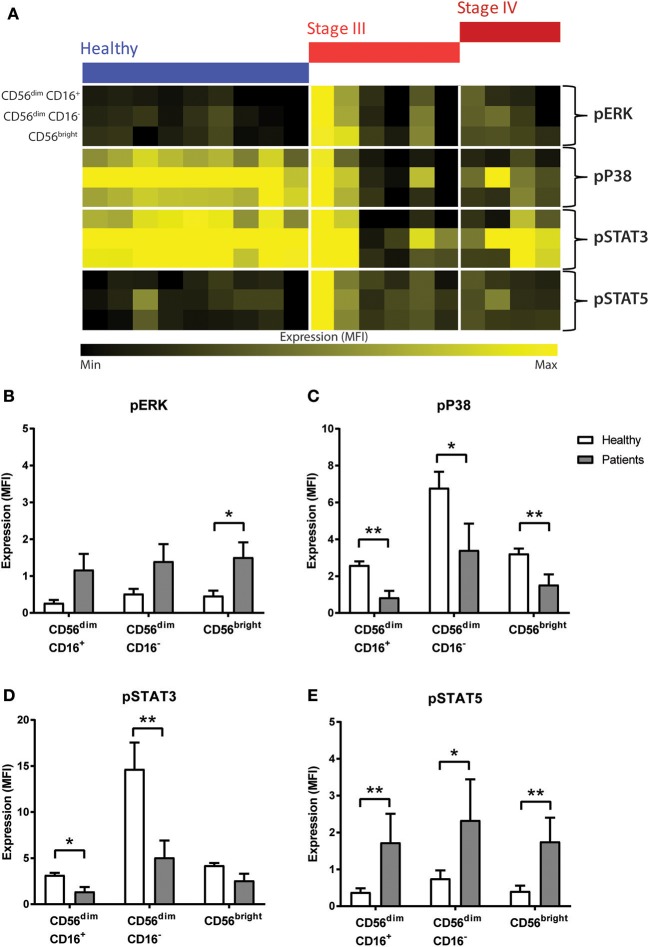
Phosphorylation signalling responses in NK cells of CRC patients compared to healthy volunteers. **(A)** The median fluorescence intensity (MFI) of pERK, pP38, pSTAT3, and pSTAT5 in the NK cell sub-population between the healthy and the CRC patients were visually examined in a heatmap. The difference in the expression of **(B)** pERK, **(C)** pP38, **(D)** pSTAT3, and **(E)** pSTAT5 in the NK cell sub-populations between the CRC patients and healthy volunteers was statistically analysed using Mann-Whiteny *U*-test. Data presented as mean ± SEM. ^*^p < 0.05 ^**^p < 0.01. *n* = 19.

No statistical difference in pP65, pAKT, pMAPKAPK2, or pSTAT1 was seen between people with CRC and the healthy volunteers (Supplementary Figure 6). However, results showed significant changes in pERK, pP38, pSTAT3, and pSTAT5 between the people with CRC at baseline and healthy volunteers (Figure 4A). The heatmap showed a global increase in pERK, and pSTAT5 but a decrease in pP38 and pSTAT3 in both the stage III and stage IV people with CRC compared to healthy.

Statistical analysis confirmed this observation as an increase was seen in pERK across all the NK cell sub-population, and this reached significance in the CD56^bright^ sub-population (*p* = 0.0277; Figure 4B). Similarly, results showed a significant decrease in pP38 in CD56^dim^ CD16^+^ (*p* = 0.0028), CD56^dim^ CD16^−^ (*p* = 0.0172), and CD56^bright^ sub-populations (*p* = 0.0041; Figure 4C).

Previous literature have demonstrated that NK cell development and survival is highly dependent on cytokines such as IL-2, IL-12, IL-15, IL-18, and IL-21, most of which signal through the JAK-STAT signalling pathway (23). A statistical analysis of the JAK/STAT signalling pathways found a significant decrease in pSTAT3 in CD56^dim^ CD16^+^ and CD56^dim^ CD16^−^ (*p* = 0.0219 and *p* = 0.0030, respectively, Figure 4D) and a significant increase in pSTAT5 across all 3 NK cell subtypes in CRC patients prior to undergoing chemotherapy (*p* = 0.0044, *p* = 0.0330, and *p* = 0.0021; Figure 4E).

## Discussion

We have used mass cytometry to enhance our understanding of the immune profile of advanced CRC patients undergoing standard cytotoxic chemotherapy. The unsupervised clustering analysis of our high-dimensional data following an acute dose of chemotherapy revealed a decrease in cluster 14, identified as NK cells. Further investigation of the NK cells using manual gating found a distinct and opposing shift in the NK cell subtypes with a decrease in CD56^dim^ CD16^+^ population to CD56^dim^ CD16^−^ and CD56^bright^ population throughout the long-term chemotherapy, particularly at the later cycles of the treatment. Furthermore, we analysed the phosphorylation status of various signalling proteins and found an increase in pERK and pSTAT5 and a decrease in pP38 and pSTAT3 universally in all NK sub-populations of people with CRC compared to healthy volunteers.

The technological development in high dimensional data, including mass cytometry, has led to a surge in the development of unsupervised, automated clustering analysis. A study by Weber and Robinson (15) compared the clustering methods for high dimensional data and recommended FlowSOM, an R package from Bioconductor, as the first-choice clustering analysis as they found that it gave the best performance with a fast run-time. Therefore, we chose to analyse our data using FlowSOM. This clustering algorithm analyses data using self-organising maps (SOM), followed by hierarchical consensus meta-cluster to merge clusters (15, 20). The use of the clustering analysis of our high dimensional data allowed the rapid assessment of the effect of acute chemotherapy and identified a unique and under-appreciated finding that NK cells as the immune populations that were most affected by the cytotoxic treatment. Chemotherapy is known to induce cell death through apoptosis as well as other non-apoptotic death such as necrosis, autophagy, mitotic catastrophe, and senescence (24). Myeloid cells, granulocytes, and platelets are known to be reduced following cytotoxic chemotherapy and leucopoenia, neutropenia and thrombocytopenia are common toxicities in patients treated with FOLFOX therapy (25). However, the 14 day cycle is sufficient for repopulation of the hematopoietic stem cell population that both innate and adaptive cells, including NK cells, are derived (26). Therefore, it is interesting to note that the NK cells did not follow an equivalent cycle-dependent rebound.

NK cells are innate lymphoid cells that are involved in preventing tumours and controlling tumour growth (27). NK cells are able to directly and indirectly kill tumour cells without the requirement of prior sensitisation or antigen (28). In this study, we characterised 3 subtypes of NK cells; CD56^dim^ CD16^+^ and CD56^bright^ CD16^−^, and CD56^bright^ cells. CD56^bright^ NK cell sub-populations have low cytotoxic function but are highly proliferative and are specialised in cytokine production responsible for the activation and recruitment of macrophages and T cells into the tumour microenvironment (29, 30). In peripheral blood, CD56^bright^ cells are classified as the stage 4 of NK cell maturation and development and are considered less mature (27, 31). The CD56^bright^ population are known to transition and convert to the more mature sub-populations (stage 5) in a sequential manner, CD56^dim^ CD16^−^ cells followed by CD56^dim^ CD16^+^ NK cells which have lower proliferative ability but have high cytotoxic function (27, 31, 32). Although the CD56^dim^ population are considered more mature as they are in a higher stage of maturation and development, both CD56^bright^ and CD56^dim^ population are both functionally active and are observed to perform different but equally important functions through either cytotoxicity or secretion of cytokines to induce an immunoregulatory environment (27, 31). In CRC, NK cells have long been shown to infiltrate the tumour microenvironment and are associated with an improved prognosis (33, 34). In addition, NK cells are also important in preventing recurrence by protecting against cancer-initiating cells (35).

The most abundant subtype of NK cells (~15% of all immune cells) identified in our study were the CD56^dim^ CD16^+^ sub-population, which was not found to be statistically different in the baseline peripheral blood samples of people with CRC compared to healthy people. Similarly, the CD56^bright^ population was not statistically different in the peripheral blood of people with CRC compared to healthy people in baseline samples. In contrast, we found a statistical increase in the numbers of CD56^+^ CD16^−^ NK cells in CRC patients. CD16 is a low-affinity FcγRIII that binds antibody-coated targets and signals antibody dependent cellular cytotoxicity (ADCC) (36, 37). CD16 specifically binds to the Fc portion of the IgG antibodies on the surface of coated cells and triggers degranulation of intracellular granules, which kill the infected or tumour cells (30, 36). Previous studies have shown that in targeted therapies in CRC, such as cetuximab, NK cells contributes to ADCC and the higher frequency of NK cells predict patient response to therapy (38). The functional role of the NK cell subtypes in ADCC should be further investigated to understand the clinical relevance of the decrease in the NK cell population following cytotoxic chemotherapy.

Previous literature has shown contrasting results regarding the frequency of NK cells in the peripheral blood and tumours of CRC patients. A study by Rocca et al. (39) found no difference in the NK cell population, identified as CD3^−^ CD56^+^, in the peripheral blood of CRC patients compared to the healthy donors. Similar to our results, the study found no difference in the proportion of CD56^dim^ and CD56^bright^ in total NK cells. In contrast, the same group in 2016 found statistically higher levels of CD3^−^ CD56^+^ NK cell population in the peripheral blood of CRC patients (40). However, in both studies, the authors did not interrogate the expression of CD16 in the NK cell population.

To enhance our understanding of immune evasion during the lifetime of a cancer patient, we need to consider the impact of standard cytotoxic chemotherapy on the frequency and phenotype of immune populations that mediate these tumour promoting processes. Our results showed that an acute dose of chemotherapy (the first cycle) caused a decrease in the levels of the NK cell sub-populations, particularly the CD56^dim^ CD16^+^ and the CD56^bright^ populations. Interestingly, long-term chemotherapy caused a phenotypic shift in the NK cell sub-populations, with a decrease in CD56^dim^ CD16^+^ population and an increase in the CD56^dim^ CD16^−^ and CD56^bright^ populations. In contrast, a previous study in CRC showed that third-line treatment with the EGFR monoclonal antibody, cetuximab, was not shown to affect the frequency of CD56^+^ or CD56^+^ CD16^+^ cells in advanced CRC patients (41).

As mentioned previously, CD56^dim^ CD16^+^ population has been shown to have high cytotoxic function but low cytokine production (27). In contrast, CD56^bright^ population have low cytotoxic function but are specialised in cytokine production responsible for the activation and recruitment of macrophages and recruitment of T cells into the tumour microenvironment (29, 30). It remains unclear why the CD56^dim^ CD16^+^ population decreased throughout the chemotherapy treatment. As mentioned above chemotherapy may preferentially induce cellular cytotoxicity of the CD56^dim^ CD16^+^ population or cause inhibition of transition from CD56^bright^ and CD16^dim^ CD16^−^ to a later maturation stage with an increase in cytotoxic function (27). Alternatively, migration of the CD56^dim^ CD16^+^ population to the tumour or other sites may also be a plausible explanation for the decreased frequency of this NK cell population. However, the role of chemotherapy impacting the migration of NK cells to other tissues, including tumours, is lacking evidence and will need to be explored in animal models due to the difficulties in obtaining clinically relevant tissues samples post-chemotherapy.

The increased frequency of CD56^dim^ CD16^−^ population may be due to increased loss of CD16 expression via shedding. Activation of NK cells through cytokines, such as IL-2, IL-15, and IL-18, TNF or target cells (such as tumour cells) can also lead to shedding of CD16 (42, 43). Many of these cytokines are increased in the circulation of colorectal cancer patients (44, 45). Activation of NK cells via signalling through CD16 or NKG2D leads to an increase in the metalloprotease ADAM17 which also cleaves CD16 on NK cells (30). Studies have shown that shedding or decrease of CD16 on NK cells via cytokine or direct tumour cell interactions are a common effect of several solid cancers including oral cancer (46), ovarian cancer (47), and melanoma (48). A decrease in the expression of CD16 on NK cells consequently led to a decrease in cytotoxic function of the NK cells, particularly decrease in ADCC, as well as production of IFNγ (46, 47, 49). The change in phenotype of NK cells from CD16^+^ to CD16^−^ as well as the decrease in cytotoxic activity following contact with target cells is known as “split anergy” (50–52). Split anergy can also be induced by stimulation with IL-2 and anti-CD16 antibodies or contact with monocytes (51). Further investigation using a larger cohort of stage IV CRC patients is needed to determine the effect of the decrease in frequency and anti-tumour function of NK cell sub-populations and response to therapy and survival outcome of the patients.

In addition to assessing the levels of NK cells sub-populations, this study was able to measure the phosphorylation status of numerous inflammatory signalling responses at a single-cell level in one small clinical sample using mass cytometry. In baseline samples, results in this study found a significantly lower expression of pSTAT3 and an increase in pSTAT5 across the NK cell subtypes in CRC patients. NK cell development in highly dependent on cytokines such as IL-2, IL-12, IL-15, IL-18, and IL-21, most of which signal through the JAK-STAT signalling pathway (23). Previous literature has shown that STAT3 is a negative regulator of NK cell function (53). *In vivo* studies by Gotthardt et al. (54) showed that a loss of STAT3 in NK cells enhanced the cytolytic activity resulting in enhanced tumour surveillance. In contrast, STAT5 has shown to be vital for NK cell development and survival through signalling from IL-2 and IL-15 (55). The decrease in pSTAT3 and increase in pSTAT5 suggest that the cytokines that target the pSTAT5 signalling pathway, such as IL-2 and IL-15, might play a more vital role in the activation and function of NK cells in this cohort of advanced CRC patients over cytokines that primarily signal through the STAT3 pathway, such as IL-12 and IL-21. Additionally supporting this finding is the increase in pERK and decrease in pP38 in the NK cell sub-populations, particularly in CD56^bright^ population, in this study. Previous studies in NK cells found that IL-2 activated MAPK/ERK signalling but not the mitogen activated protein kinase (MAPK) P38 signalling pathway to regulate NK cell function (56). Interestingly, a study by Peruzzi et al. (57) also showed that IL-2 or IL-15 exposure led to the shedding of CD16 on the surface of NK cells which could also explain the decrease in CD16+ NK cells following chemotherapy.

There are important limitations to consider when using mass cytometry for discovery phase projects, such as this study. Statistical analysis of small datasets is challenging when obtaining the levels of high dimensional data obtained through a mass cytometry experiment. As this was a discovery project, we avoided using *post-hoc* corrections in our statistical analysis to reduce the risk of type II errors (false negatives) in this small cohort. However, this also increases the risk of type I error (false positives). As a recommendation following the mass cytometry discovery project, future studies need to utilise a larger cohort size and prospectively use *post-hoc* multiple comparison corrections (e.g., Bonferroni correction of pairwise comparisons or using a false discovery rate correction). Other sources of limitations were missing time points in some patients, particularly the end of infusion of day 3 sample, which could cause potential bias of results following a single acute dose of chemotherapy. Missing samples later during chemotherapy are due to unplanned discontinuation of chemotherapy for toxicity or progressive disease and would be difficult to predict when these random events occur. Finally, future experiments with mass cytometry should incorporate functional studies to validate the biological relevance of the changes in phenotype and how these are related to clinical outcomes. However, researchers do need to be mindful of the larger volumes of blood required to complete these more extensive functional assays in patients undergoing active chemotherapy and there may be challenges of acceptance by patients and ethics committees.

Despite only having access to a small number of patients, this study was nonetheless able to identify differences in the NK cell population in CRC patients undergoing standard chemotherapy treatment. Following the patients throughout their chemotherapy treatment (~5–6 months) in conjunction with use of mass cytometry proved to be a powerful combination that allowed us to profile the immune system of these patients as they underwent treatment and identify a novel phenotypic shift in the NK cell populations. Following standard cytotoxic chemotherapy, we have identified that there is an unrecognised deficiency in NK cell frequencies that needs to be considered in the therapeutic decisions and monitoring of treatment response and survival.

## Data Availability Statement

The datasets generated for this study are available on request to the corresponding author.

## Ethics Statement

This study was approved by Northern Sydney Local Health District and North Shore Private Hospital Human Ethics Committees, and written, informed consent was obtained from all patients prior to participation in the study.

## Author Contributions

DS helped with enrolment of patients, performed experiments, analysed and interpreted data, and wrote the manuscript. HM helped with design of experiments, trained and helped DS run the samples on the mass cytometer, advised on analysis and interpreted obtained data, and critically revised the manuscript. CD, NP, and SC helped with design of study, enrolment of patients, and reviewed the manuscript. SB interpreted obtained data and critically reviewed the manuscript. KC designed the study, enrolled patients, interpreted obtained data, participated in writing the manuscript, and critically reviewed the manuscript.

### Conflict of Interest

The authors declare that the research was conducted in the absence of any commercial or financial relationships that could be construed as a potential conflict of interest.

## References

[1] Favoriti P Carbone G Greco M Pirozzi F Pirozzi RE Corcione F. Worldwide burden of colorectal cancer: a review. Updates Surg. (2016) 68:7–11. 10.1007/s13304-016-0359-y 27067591

[2] Andre T Boni C Mounedji-Boudiaf L Navarro M Tabernero J Hickish T. Oxaliplatin, fluorouracil, and leucovorin as adjuvant treatment for colon cancer. N Engl J Med. (2004) 350:2343–51. 10.1056/NEJMoa032709 15175436

[3] Brenner H Kloor M Pox CP. Colorectal cancer. Lancet. (2014) 383:1490–502. 10.1016/S0140-6736(13)61649-9 24225001

[4] Sclafani F Cunningham D Cetuximab or bevacizumab in metastatic colorectal cancer? Lancet Oncol. (2014) 15:1040–1. 10.1016/S1470-2045(14)70360-2 25088941

[5] Tol J Koopman M Cats A Rodenburg CJ Creemers GJM Schrama JG. Chemotherapy, bevacizumab, and cetuximab in metastatic colorectal cancer. N Engl J Med. (2009) 360:563–72. 10.1056/NEJMoa0808268 19196673

[6] Hanahan D Weinberg RA. Hallmarks of cancer: the next generation. Cell. (2011) 144:646–74. 10.1016/j.cell.2011.02.013 21376230

[7] Wang K Karin M. Tumor-elicited inflammation and colorectal cancer. Adv Cancer Res. (2015) 128:173–96. 10.1016/bs.acr.2015.04.014 26216633

[8] Albini A Tosetti F Benelli R Noonan DM. Tumor inflammatory angiogenesis and its chemoprevention. Cancer Res. (2005) 65:10637–41. 10.1158/0008-5472.CAN-05-3473 16322203

[9] Hu T Li LF Shen J Zhang L Cho CH. Chronic inflammation and colorectal cancer: the role of vascular endothelial growth factor. Curr Pharm Des. (2015) 21:2960–7. 10.2174/1381612821666150514104244 26004415

[10] Overman MJ Ernstoff MS Morse MA. Where we stand with immunotherapy in colorectal cancer: deficient mismatch repair, proficient mismatch repair, and toxicity management. Am Soc Clin Oncol Educ Book. (2018) 38:239–47. 10.1200/EDBK_200821 30231358

[11] Le DT Uram JN Wang H Bartlett BR Kemberling H Eyring AD. PD-1 blockade in tumors with mismatch-repair deficiency. N Engl J Med. (2015) 372:2509–20. 10.1056/NEJMoa1500596 26028255 PMC4481136

[12] Overman MJ McDermott R Leach JL Lonardi S Lenz HJ Morse MA. Nivolumab in patients with metastatic DNA mismatch repair-deficient or microsatellite instability-high colorectal cancer (CheckMate 142): an open-label, multicentre, phase 2 study. Lancet Oncol. (2017) 18:1182–91. 10.1016/S1470-2045(17)30422-9 28734759 PMC6207072

[13] Tanner SD Baranov VI Ornatsky OI Bandura DR George TC. An introduction to mass cytometry: fundamentals and applications. Cancer Immunol Immunother. (2013) 62:955–65. 10.1007/s00262-013-1416-8 23564178 PMC11029414

[14] Nair N Mei HE Chen SY Hale M Nolan GP Maecker HT. Mass cytometry as a platform for the discovery of cellular biomarkers to guide effective rheumatic disease therapy. Arthritis Res Ther. (2015) 17:127. 10.1186/s13075-015-0644-z 25981462 PMC4436107

[15] Weber LM Robinson MD. Comparison of clustering methods for high-dimensional single-cell flow and mass cytometry data. Cytometry A. (2016) 89:1084–96. 10.1002/cyto.a.23030 27992111

[16] eviQ Colorectal Adjuvant FOLFOX6 (Modified) (Fluorouracil Leucovorin Oxaliplatin). eviQ Cancer Treatments Online, Cancer Institute NSW (2018). Available online at: https://www.eviq.org.au/medical-oncology/colorectal/adjuvant-and-neoadjuvant/637-colorectal-adjuvant-folfox6-modified-fluoro (accessed April 1, 2019).

[17] Shinko D Ashhurst TM McGuire HM Charles KA. Staining of phosphorylated signalling markers protocol for mass cytometry. Methods Mol Biol. (2019) 1989:139–46. 10.1007/978-1-4939-9454-0_10 31077104

[18] Ashhurst TM Cytometry Analysis Pipeline for large and compleX Datasets v2.4. GitHub repository (2018). Available online at: https://github.com/sydneycytometry/CAPX

[19] Ashhurst TM Cox DA Smith AL King NJC. Analysis of the murine bone marrow hematopoietic system using mass and flow cytometry. Methods Mol Biol. (2019) 1989:159–92. 10.1007/978-1-4939-9454-0_12 31077106

[20] Van Gassen S Callebaut B Van Helden MJ Lambrecht BN Demeester P Dhaene T. FlowSOM: using self-organizing maps for visualization and interpretation of cytometry data. Cytometry A. (2015) 87:636–45. 10.1002/cyto.a.22625 25573116

[21] Mantovani A Allavena P Sica A Balkwill F. Cancer-related inflammation. Nature. (2008) 454:436–44. 10.1038/nature07205 18650914

[22] Shinko D Diakos CI Clarke SJ Charles KA. Cancer-related systemic inflammation: the challenges and therapeutic opportunities for personalized medicine. Clin Pharmacol Ther. (2017) 102:599–610. 10.1002/cpt.789 28699186

[23] Di Santo JP. Natural killer cell developmental pathways: a question of balance. Annu Rev Immunol. (2006) 24:257–86. 10.1146/annurev.immunol.24.021605.090700 16551250

[24] Ricci MS Zong W-X. Chemotherapeutic approaches for targeting cell death pathways. Oncol. (2006) 11:342–57. 10.1634/theoncologist.11-4-342 16614230 PMC3132471

[25] Hochster HS Hart LL Ramanathan RK Childs BH Hainsworth JD Cohn AL. Safety and efficacy of oxaliplatin and fluoropyrimidine regimens with or without bevacizumab as first-line treatment of metastatic colorectal cancer: results of the TREE Study. J Clin Oncol. (2008) 26:3523–9. 10.1200/JCO.2007.15.4138 18640933

[26] Randall TD Weissman IL. Phenotypic and functional changes induced at the clonal level in hematopoietic stem cells after 5-fluorouracil treatment. Blood. (1997) 89:3596–606. 10.1182/blood.V89.10.3596.3596_3596_3606 9160664

[27] Abel AM Yang C Thakar MS Malarkannan S. Natural killer cells: development, maturation, and clinical utilization. Front Immunol. (2018) 9:1869. 10.3389/fimmu.2018.01869 30150991 PMC6099181

[28] Nicholson SE Keating N Belz GT. Natural killer cells and anti-tumor immunity. Mol Immunol. (2017) 110:40–7. 10.1016/j.molimm.2017.12.002 29233542

[29] Freud AG Mundy-Bosse BL Yu J Caligiuri MA. The broad spectrum of human natural killer cell diversity. Immunity. (2017) 47:820–33. 10.1016/j.immuni.2017.10.008 29166586 PMC5728700

[30] Romee R Foley B Lenvik T Wang Y Zhang B Ankarlo D. NK cell CD16 surface expression and function is regulated by a disintegrin and metalloprotease-17 (ADAM17). Blood. (2013) 121:3599–608. 10.1182/blood-2012-04-425397 23487023 PMC3643761

[31] Poli A Michel T Theresine M Andres E Hentges F Zimmer J. CD56bright natural killer (NK) cells: an important NK cell subset. Immunology. (2009) 126:458–65. 10.1111/j.1365-2567.2008.03027.x 19278419 PMC2673358

[32] Jacobs R Hintzen G Kemper A Beul K Kempf S Behrens G. CD56bright cells differ in their KIR repertoire and cytotoxic features from CD56dim NK cells. Eur J Immunol. (2001) 31:3121–6. 10.1002/1521-4141(2001010)31:10<3121::AID-IMMU3121>3.0.CO;2-4 11592089

[33] Coca S Perez-Piqueras J Martinez D Colmenarejo A Saez MA Vallejo C. The prognostic significance of intratumoral natural killer cells in patients with colorectal carcinoma. Cancer. (1997) 79:2320–8. 10.1002/(SICI)1097-0142(19970615)79:12<2320::AID-CNCR5>3.0.CO;2-P 9191519

[34] Donadon M Hudspeth K Cimino M Di Tommaso L Preti M Tentorio P. Increased infiltration of natural killer and t cells in colorectal liver metastases improves patient overall survival. J Gastrointest Surg. (2017) 21:1226–36. 10.1007/s11605-017-3446-6 28536806

[35] Tallerico R Todaro M Di Franco S Maccalli C Garofalo C Sottile R. Human NK cells selective targeting of colon cancer-initiating cells: a role for natural cytotoxicity receptors and MHC class I molecules. J Immunol. (2013) 190:2381–90. 10.4049/jimmunol.1201542 23345327

[36] Cooper MA Caligiuri MA Isolation and characterization of human natural killer cell subsets. Curr Protoc Immunol. (2004) 60:7.34.1–12. 10.1002/0471142735.im0734s60 18432933

[37] Leibson PJ. Signal transduction during natural killer cell activation: inside the mind of a killer. Immunity. (1997) 6:655–61. 10.1016/S1074-7613(00)80441-0 9208838

[38] Trotta AM Ottaiano A Romano C Nasti G Nappi A De Divitiis C. Prospective evaluation of cetuximab-mediated antibody-dependent cell cytotoxicity in metastatic colorectal cancer patients predicts treatment efficacy. Cancer Immunol Res. (2016) 4:366–74. 10.1158/2326-6066.CIR-15-0184 26817995

[39] Rocca YS Roberti MP Arriaga JM Amat M Bruno L Pampena MB. Altered phenotype in peripheral blood and tumor-associated NK cells from colorectal cancer patients. Innate Immun. (2013) 19:76–85. 10.1177/1753425912453187 22781631

[40] Rocca YS Roberti MP Julia EP Pampena MB Bruno L Rivero S Phenotypic and functional dysregulated blood NK cells in colorectal cancer patients can be activated by cetuximab plus IL-2 or IL-15. Front Immunol. (2016) 7:413 10.3389/fimmu.2016.00413 27777574 PMC5056190

[41] Matic IZ Kolundzija B Damjanovic A Spasic J Radosavljevic D Dordic Crnogorac M. Peripheral white blood cell subsets in metastatic colorectal cancer patients treated with cetuximab: the potential clinical relevance. Front Immunol. (2017) 8:1886. 10.3389/fimmu.2017.01886 29354119 PMC5758541

[42] Felce JH Dustin ML. Natural killers shed attachments to kill again. J Cell Biol. (2018) 217:2983. 10.1083/jcb.201807105 30108125 PMC6122983

[43] Srpan K Ambrose A Karampatzakis A Saeed M Cartwright ANR Guldevall K. Shedding of CD16 disassembles the NK cell immune synapse and boosts serial engagement of target cells. J Cell Biol. (2018) 217:3267. 10.1083/jcb.201712085 29967280 PMC6122987

[44] Mager LF Wasmer M-H Rau TT Krebs P. Cytokine-induced modulation of colorectal cancer. Front Oncol. (2016) 6:96. 10.3389/fonc.2016.00096 27148488 PMC4835502

[45] Yamaguchi M Okamura S Yamaji T Iwasaki M Tsugane S Shetty V. Plasma cytokine levels and the presence of colorectal cancer. PLoS ONE. (2019) 14:e0213602. 10.1371/journal.pone.0213602 30883594 PMC6422333

[46] Jewett A Cacalano NA Head C Teruel A. Coengagement of CD16 and CD94 receptors mediates secretion of chemokines and induces apoptotic death of naive natural killer cells. Clin Cancer Res. (2006) 12:1994–2003. 10.1158/1078-0432.CCR-05-2306 16609008

[47] Lai P Rabinowich H Crowley-Nowick PA Bell MC Mantovani G Whiteside TL. Alterations in expression and function of signal-transducing proteins in tumor-associated T and natural killer cells in patients with ovarian carcinoma. Clin Cancer Res. (1996) 2:161–73. 9816103

[48] Vujanovic L Chuckran C Lin Y Ding F Sander CA Santos PM. CD56dim CD16– natural killer cell profiling in melanoma patients receiving a cancer vaccine and interferon-α. Front Immunol. (2019) 10:14. 10.3389/fimmu.2019.00014 30761123 PMC6361792

[49] Jewett A Cavalcanti M Bonavida B. Pivotal role of endogenous TNF-alpha in the induction of functional inactivation and apoptosis in NK cells. J Immunol. (1997) 159:4815–22. 9366406

[50] Belyaev NN Abramova VA. Transmission of “split anergy” from tumor infiltrating to peripheral NK cells in a manner similar to “infectious tolerance”. Med Hypotheses. (2014) 82:129–33. 10.1016/j.mehy.2013.11.019 24332531

[51] Jewett A Arasteh A Tseng HC Behel A Arasteh H Yang W. Strategies to rescue mesenchymal stem cells (MSCs) and dental pulp stem cells (DPSCs) from NK cell mediated cytotoxicity. PLoS ONE. (2010) 5:e9874. 10.1371/journal.pone.0009874 20360990 PMC2847602

[52] Jewett A Tseng H-C. Potential rescue, survival and differentiation of cancer stem cells and primary non-transformed stem cells by monocyte-induced split anergy in natural killer cells. Cancer Immunol Immunother. (2012) 61:265–74. 10.1007/s00262-011-1163-7 22116348 PMC11029795

[53] Cacalano NA. Regulation of natural killer cell function by STAT3. Front Immunol. (2016) 7:128. 10.3389/fimmu.2016.00128 27148255 PMC4827001

[54] Gotthardt D Putz EM Straka E Kudweis P Biaggio M Poli V. Loss of STAT3 in murine NK cells enhances NK cell-dependent tumor surveillance. Blood. (2014) 124:2370–9. 10.1182/blood-2014-03-564450 25185262

[55] Gotthardt D Putz EM Grundschober E Prchal-Murphy M Straka E Kudweis P. STAT5 is a key regulator in NK cells and acts as a molecular switch from tumor surveillance to tumor promotion. Cancer Discov. (2016) 6:414–29. 10.1158/2159-8290.CD-15-0732 26873347

[56] Yu TK Caudell EG Smid C Grimm EA IL-2 activation of NK cells: involvement of MKK1/2/ERK but not p38 kinase pathway. J Immunol. (2000) 164:6244–51. 10.4049/jimmunol.164.12.6244 10843677

[57] Peruzzi G Femnou L Gil-Krzewska A Borrego F Weck J Krzewski K. Membrane-type 6 matrix metalloproteinase regulates the activation-induced downmodulation of CD16 in human primary NK cells. J Immunol. (2013) 191:1883–94. 10.4049/jimmunol.1300313 23851692 PMC3745217

