# Supplementary material for: Mass Cytometry Reveals a Sustained Reduction in CD16+ Natural Killer Cells Following Chemotherapy in Colorectal Cancer Patients
Source: Front Immunol. 2019 Nov 5;10:2584. doi: 10.3389/fimmu.2019.02584 (PMC6848231; doi:10.3389/fimmu.2019.02584)
Supplement: Supplementary file 1 [file Data_Sheet_1.docx]

# **Supplementary Material**

**Supplementary table 1.** The antibody panel used for mass cytometry.

| **Metal Tag** | **Target** | **Clone** | **Antibody supplier** |
| --- | --- | --- | --- |
| 141Pr | CD235ab | HIR2 | Fluidigm* |
| 142Nd | CD19 | HIB19 | RFHSB^§^ |
| 143Nd | CD33 | WM53 | Biolegend^#^ |
| 144Nd | CD11b | ICR544 | Biolegend |
| 145Nd | CD4 | RPA-T4 | Biolegend |
| 146Nd | CD8 | RPA-T8 | RFHSB |
| 147Sm | CD7 | CD7-6B7 | Fluidigm |
| 148Sm | CD16 | 3G8 | RFHSB |
| 149Sm | CD25 | 2A3 | RFHSB |
| 150Nd | pSTAT5 (Y694) | 47 | Fluidigm |
| 151Eu | CD123 | 6H6 | RFHSB |
| 152Sm | CD66b | 80H3 | Fluidigm |
| 153Eu | pSTAT1 (Y701) | 58D6 | Fluidigm |
| 154Sm | pAKT | J1-223.371 | BD^ɸ^ |
| 155Gd | CD27 | L128 | RFHSB |
| 156Gd | pP38 (T180/Y182) | 36/p38 | BD |
| 158Gd | pSTAT3 (Y705) | 4/p | Fluidigm |
| 159Tb | CD11c | Bu15 | RFHSB |
| 160Gd | CD14 | M5E2 | RFHSB |
| 161Dy | CD69 | FN50 | Biolegend |
| 162Dy | FoxP3 | PCH101 | RFHSB |
| 163Dy | CD56 (NCAM) | HCD56 | Biolegend |
| 164Dy | CD45RO | UCHL1 | RFHSB |
| 166Er | pP65 (S529) | K10.895.12.50 | BD |
| 167Er | CD197 (CCR7) | G043H7 | Biolegend |
| 168Er | pERK (T202/Y204) | D13.14.4E | CST^†^ |
| 169Tm | CD45RA | HI100 | RFHSB |
| 170Er | CD3 | UCHT1 | RFHSB |
| 171Yb | CD66a/c/e | ASL-32 | Biolegend |
| 172Yb | CD130 (gp130) | 2E1B02 | Biolegend |
| 173Yb | pMAPKAPK2 (Thr334) | 27B7 | CST |
| 174Yb | HLA-DR | L243 | RFHSB |
| 175Lu | Arginase 1 | 658922 | R&D^//^ |
| 176Yb | CD127 (IL-7Rα) | A019D5 | RFHSB |

* Fluidigm, San Francisco, California

§ RFHSB = Ramaciotti Facility for Human Systems Biology, University of Sydney

# Biolegend, San Diego, California

ɸ BD Biosciences, San Jose, California

† CST = Cell Signalling Technology, Danvers, Massachusetts

// R&D Systems, Minneapolis, Minnesota


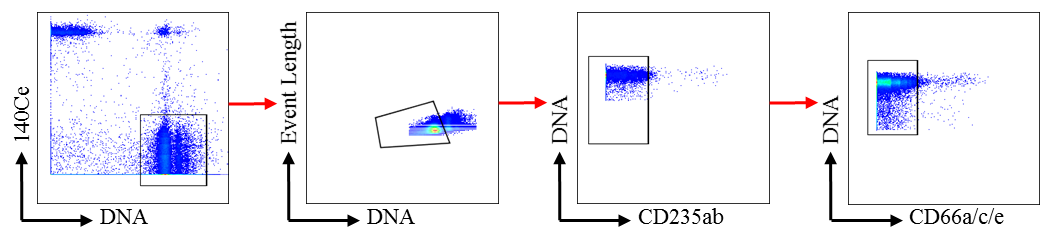


**Supplementary Figure 1.** The background gating strategy used for all manual gating analysis. The cells were identified as DNA^+^ 140Ce^-^. The single cells were then gated from the DNA vs Event Length gate followed by gating the CD235ab^-^ population to eliminate any red blood cells and the CD66a/c/e^-^ population to eliminate any granulocytes.


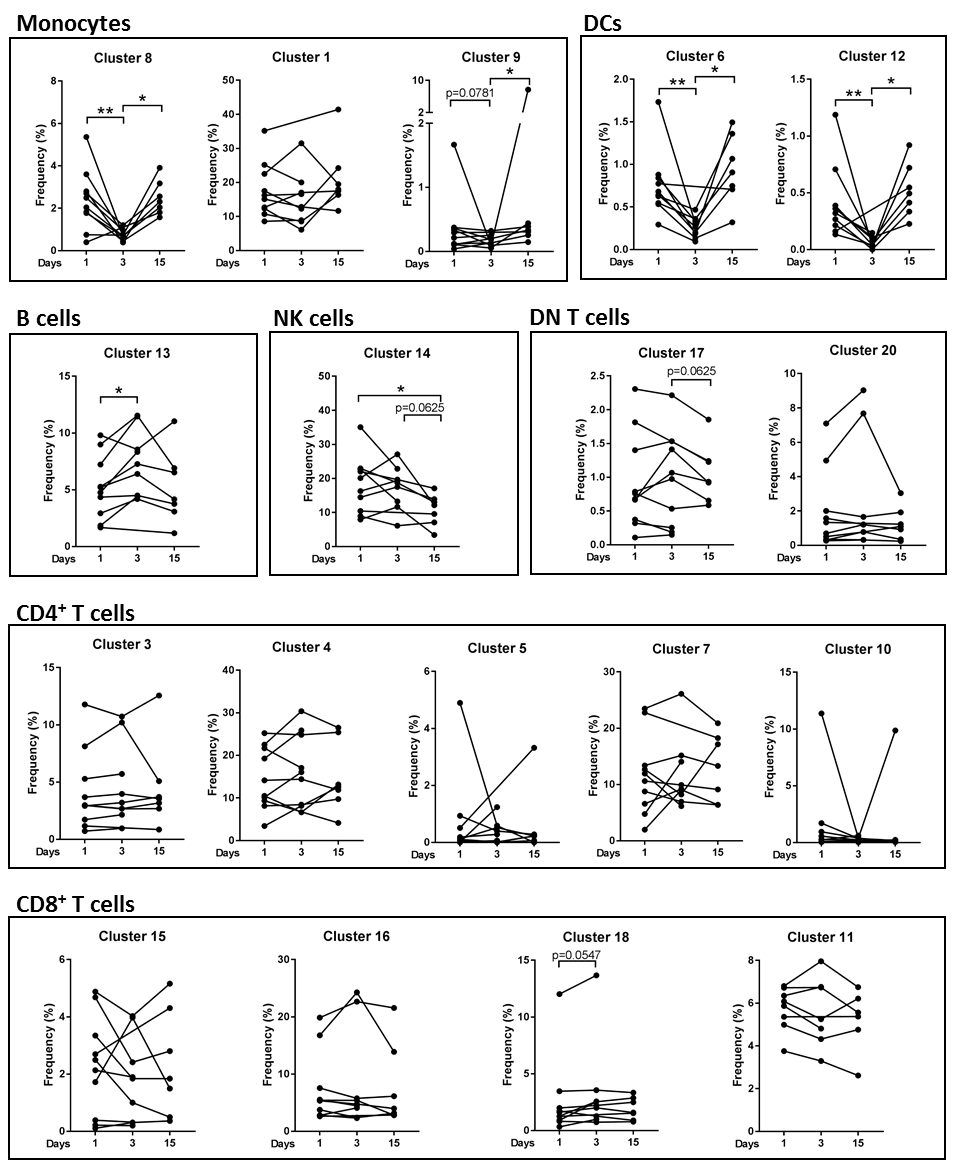


**Supplementary Figure 2.** Effect of acute chemotherapy on clusters identified by FLOWSOM analysis. PBMCs from healthy volunteers and CRC patients prior to undergoing chemotherapy (baseline) and throughout chemotherapy treatment were stained with a 35-antibody mass cytometry panel and ran on the mass cytometer, Helios^TM^. The absolute number of clusters was measured after one cycle of chemotherapy (days 1, 3 and 15). Data was analysed using Wilcoxon test. DCs = Dendritic cells, NK cells = Natural killer cells, DN= Double negative. * p < 0.05, ** p<0.01. n=10.


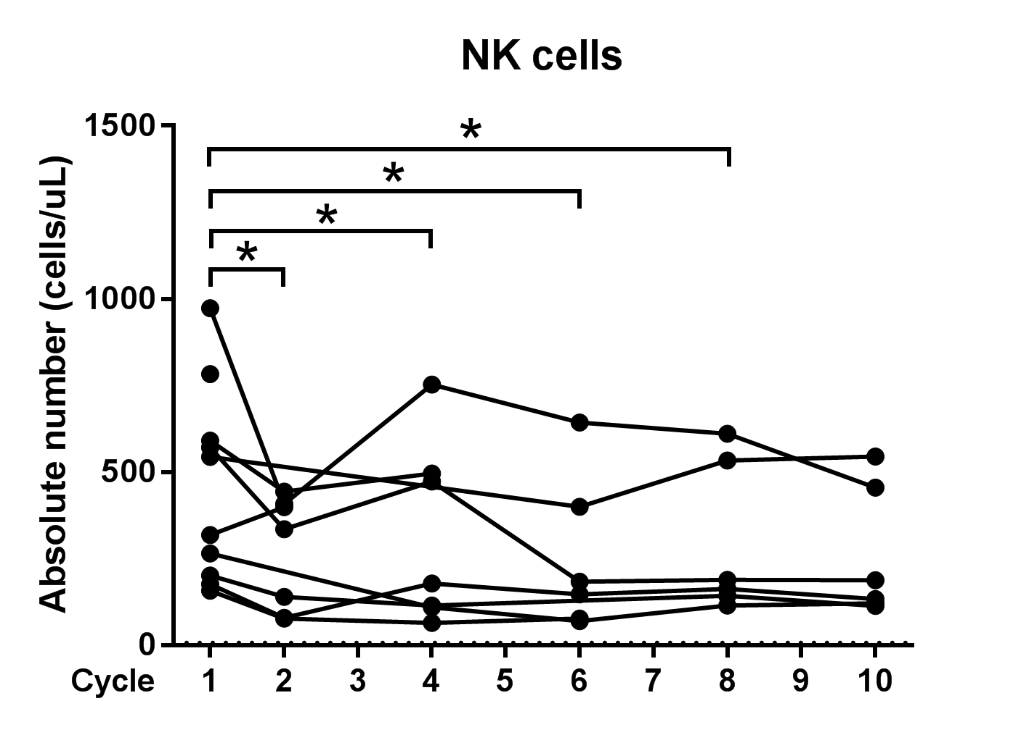

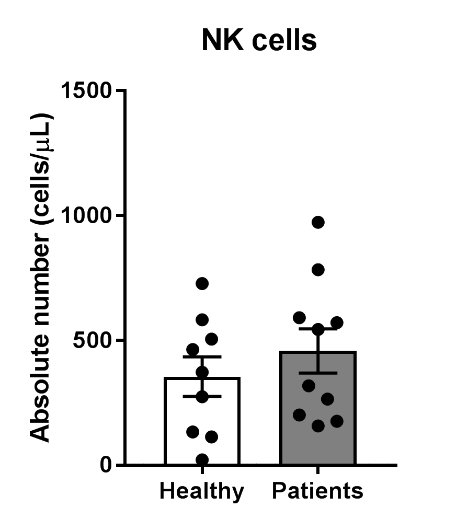

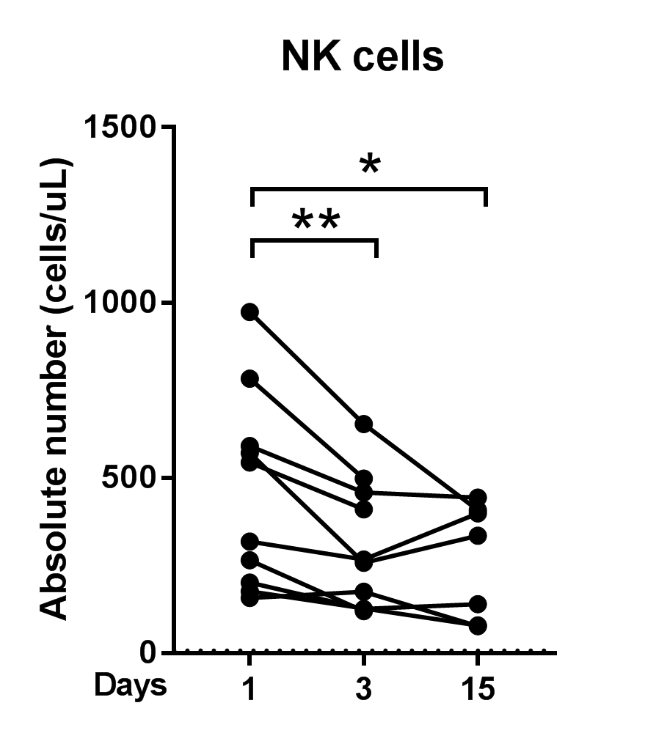


**C**

**B**

**A**

**A**

**Supplementary Figure 3.** Differences in total NK cell populations in CRC patients compared to healthy volunteers and throughout chemotherapy. PBMCs from healthy volunteers and CRC patients prior to undergoing chemotherapy (baseline) and throughout chemotherapy treatment were stained with a 35-antibody mass cytometry panel and ran on the mass cytometer, Helios^TM^. **(A)** The difference in the absolute number of total NK cells between CRC patients at baseline compared to healthy volunteers was measured and analysed using Mann-Whitney U test. Data presented as mean±SEM. The absolute number of total NK cells was measured after **(B)** one cycle of chemotherapy (days 1, 3 and 15) and **(C)** throughout the subsequent cycles of chemotherapy. The effect of chemotherapy on NK cell data was analysed using Wilcoxon test. NK cells = Natural killer cells. * p < 0.05, ** p<0.01. n=19.


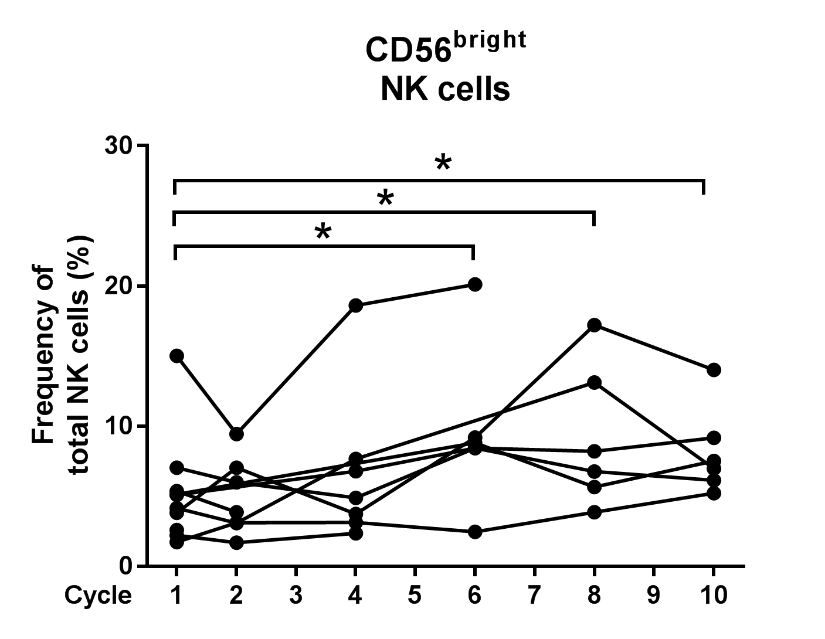

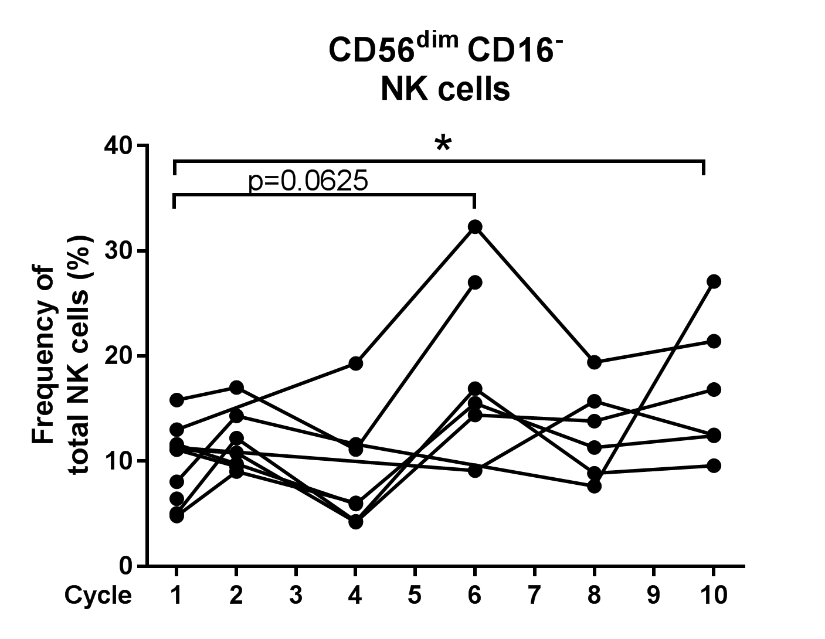

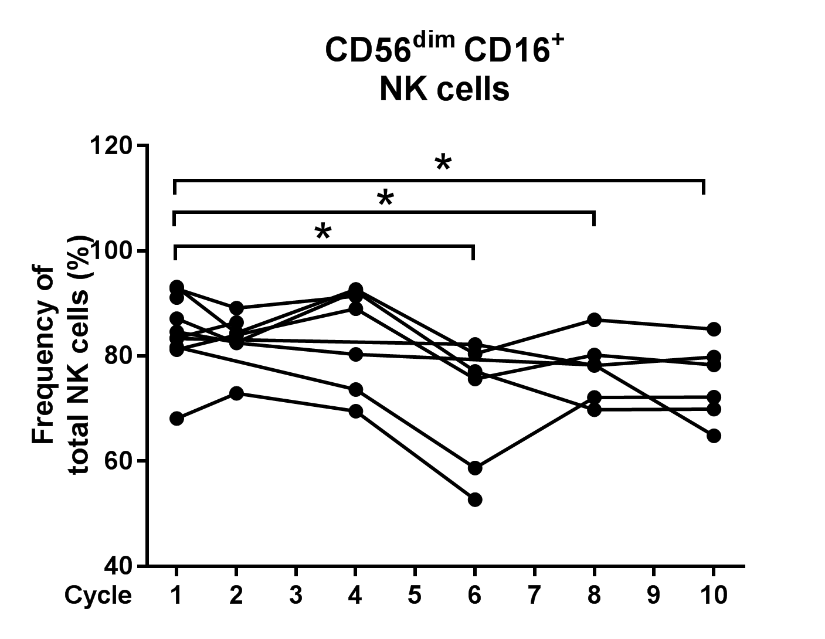


**A**

**A**

**B**

**C**

**Supplementary Figure 4.** Effect of chemotherapy on frequency of NK cell subpopulations. PBMCs from CRC patients undergoing FOLFOX chemotherapy from cycles 1-10 were collected to test the long term effect of chemotherapy. PBMCs were isolated, stained with a 35-antibody mass cytometry panel and ran on the mass cytometer, Helios^TM^. The frequency of **(A)** CD56^dim^ CD16^+^ NK cells, **(B)** CD56^dim^ CD16^-^ NK cells, and **(C)** CD56^bright^ NK cells as a percentage of total NK cells were measured throughout 10 cycles of chemotherapy and analysed using Wilcoxon test. NK cells = Natural killer cells. * p < 0.05. n=10.


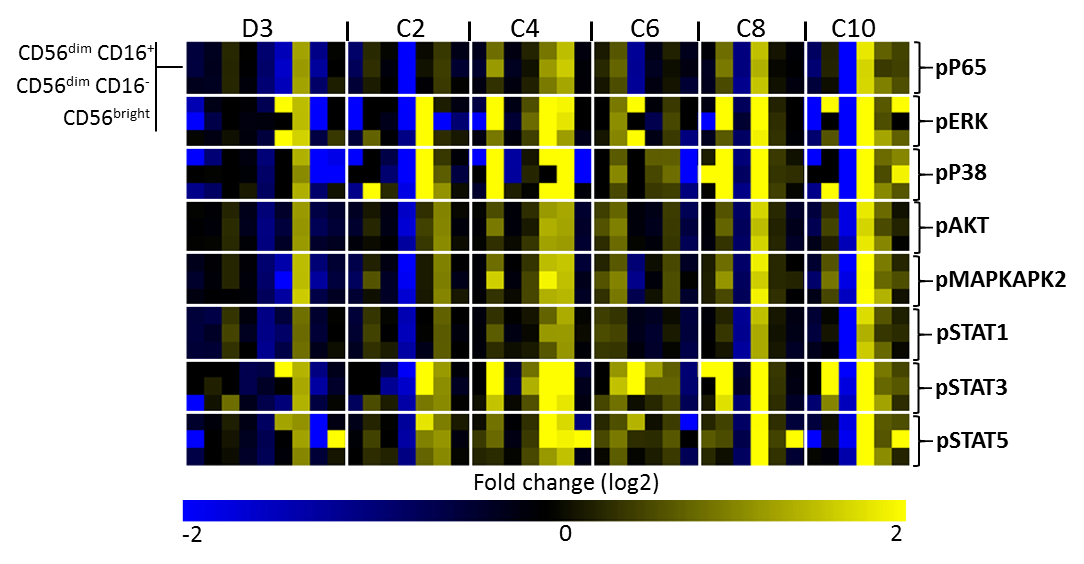


**Supplementary Figure 5.** Effect of chemotherapy on the activation of signalling responses in NK cell subpopulations. Blood samples were collected from CRC patients undergoing FOLFOX chemotherapy. Twenty millilitres of blood was collected from patients on day 1 (pre-treatment), day 3 and day 15. Blood samples from later cycles were also collected. PBMCs were isolated, stained with a 35-antibody panel and ran on the mass cytometer, Helios^TM^. The fold change in the expression of pP65, pERK, pP38, pAKT, pMAPKAPK2, pSTAT1, pSTAT3, and pSTAT5 over the baseline (day 1) for each timepoint of a patient (column) in each NK cell subpopulation (row) was examined using a heatmap. n=19.


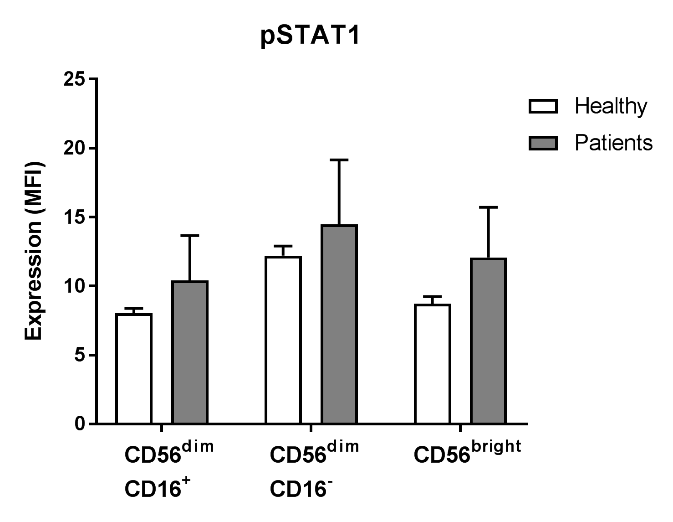

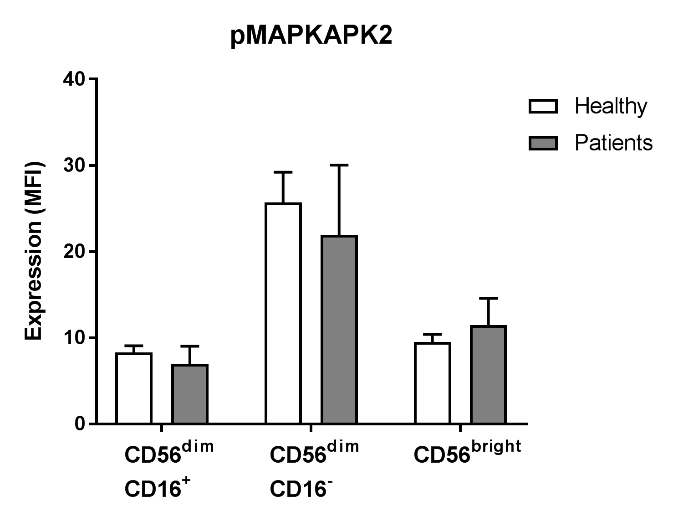

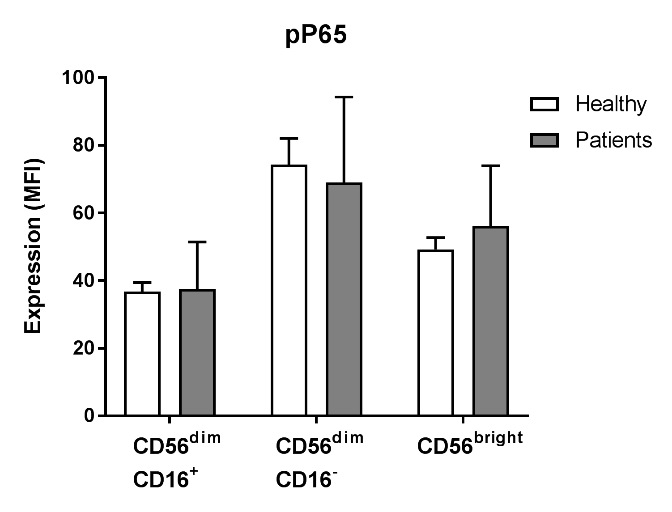

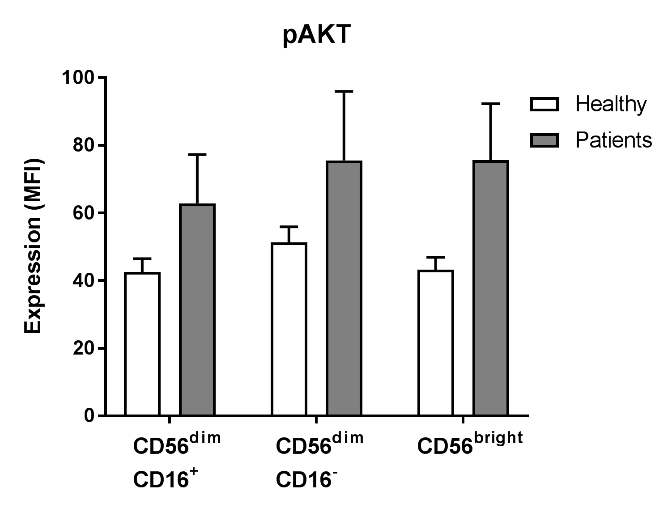


**A**

**B**

**A**

**A**

**D**

**C**

**Supplementary Figure 6.** Expression of pP65, pAKT, pMAPKAPK2, and pSTAT1 in the NK cell subpopulations. PBMCs from healthy volunteers and CRC patients prior to undergoing chemotherapy (baseline) were stained with a 35-antibody mass cytometry panel and ran on the mass cytometer, Helios^TM^. The difference in the expression of **(A)** pP65, **(B)** pAKT, **(C)** pMAPKAPK2, and **(D)** pSTAT1 in the NK cell subopulations between the CRC patients and the healthy volunteers was measured and statistically analysed using Mann-Whiteny U test. Data presented as mean±SEM. n=19.
